# Eating Behaviors of Older Adults Participating in Government-Sponsored Programs with Different Demographic Backgrounds

**DOI:** 10.5539/gjhs.v4n6p204

**Published:** 2012-10-25

**Authors:** Shahla Wunderlich, Joseph Brusca, Marti Johnson-Austin, Yeon Bai, Michelle O’ Malley

**Affiliations:** 1 Department of Health and Nutrition Sciences, Montclair State University, Montclair, NJ, USA

**Keywords:** food behaviors, older adults, high risk, nutrition programs, nutrition screening initiative

## Abstract

The purpose of this study was to determine the food behaviors of nutritionally high-risk seniors as a function of their racial background, gender, marital status, and education level. A total of 69 seniors were identified to be at high nutritional risk using the Nutrition Screening Initiative (NSI) checklist. A supplemental questionnaire (SQ) was created to examine the risk factors in relation to the participant’s demographic background. Key results indicated that Asians practiced healthy food behaviors and women were more likely to eat alone (p≤0.05). Married participants (90.9%) were most likely to consume 2 meals or more each day. College educated individuals practiced healthier eating, eating 5 servings or more of fruits and vegetables (p≤0.01) and 2 or more servings of milk and milk products (p≤0.01). These preliminary findings indicate that more studies should be conducted to focus on the demographic characteristics and food behaviors among older populations.

## 1. Background

### 1.1 A Growing Concern

It is estimated by the Administration on Aging that by 2020, people who are 65 and older will account for approximately 15.8% of the population in the United States (Administration on Aging, 2011). At a time when the aging population is on the rise, there is even more urgency to determine the socioeconomic and physiological factors that contribute to high nutritional risk among older adults. The state of nutritional risk, including factors such as food insufficiency and/or low micronutrient intake, if not corrected in time, may lead to poor health and lower quality of life (Sahyoun & Basiotis, 2001; Kim, Tse, & Boudreu, 2002). This in turn can create pain and suffering for the individuals as well as burdens on society and the medical system. Therefore, determining the seniors who are at risk of malnutrition is paramount to effective prevention and optimal nutrition and wellness interventions. These interventions, such as nutrition education and nutrition counseling combined with physical activity can be very cost effective (Wunderlich et al, 2010). The annual Medicare long term care (LTC) expenses per beneficiary grew from $5370 in 2002 to $7064 in 2005. In addition, Medicare spending for LTC as a percentage of national healthcare expenditure is projected to grow from 17.2% in 2005 to 20.7% in 2017 (Medicare Spending and Financing, 2008). Evaluating the food behaviors among different demographic groups provides some understanding of how to tailor appropriate nutrition education and counseling for each group and can improve health and wellbeing of seniors.

### 1.2 Risk Factors amongst Seniors

Older adults experience many obstacles that may prevent them from meeting their nutrition needs and keeping their health optimal. Among them are socioeconomic factors, which are important contributing factors in causing nutritional risks among older adults. Socioeconomic factors such as income level have been shown to play a significant role in the dietary behaviors of seniors. Previous studies have determined that people with lower incomes “ate less fruit, vegetables, milk, meat, poultry, and fish than high-income adults”. They also consumed less of the Adequate Intake of Nutrients than people with higher incomes (Bowman, 2007). In addition to income, there are other socioeconomic factors, which can play a role in whether a senior citizen’s diet is nutritionally sound. These factors include inadequate methods of transportation to food sources as well as lack of social capital (fear of being attacked or perceived discrimination), which can inhibit the ability to purchase healthy food. Social isolation and a low level of social support such as not being married have also been shown to increase nutritional risk (Locher Ritchie, Roth, Baker, Bodner, & Allman, 2005).

Eating behaviors are largely determined by cultural, social, and psychological factors (Elsner, 2002; Wright, Hickson, & Frost, 2006). The elderly are in a period of great transition as their social environments are changing due to marital issues (divorce or widowhood) or shifts in household environments such as moving into a setting with more or fewer people. These changes in their living circumstances, loss of mobility, or being alone may make it difficult for these individuals to maintain their normal eating habits.

Demographic factors such as gender must also be considered. Studies have shown that gender can be indicative of nutritional risk among seniors (Quandt & Chao, 2000). Gender can also be indicative of different eating behaviors. For example, a study in the European Journal of Clinical Nutrition showed that fruit and vegetable consumption was consistently based on income and education for women, while these factors did not influence men’s fruit and vegetable intake (Lallukka, Pitkaniemi, Rahkonen, Roos, Laaksonen, & Lahelma, 2010).

Racial backgrounds can also play a role in nutritional risk levels (Weatherspoon, Worthen, & Handu 2004). Race has also been shown to be an indicator for inadequate levels of nutrients such as folate and vitamin B12 in the elderly (Kim, Tse, & Boudreu, 2002). Eating habits and behaviors have also been shown to have a relationship to people’s racial backgrounds. The Third National Health and Nutrition Examination Survey from the American Dietetic Association revealed that African Americans often consumed fewer salads and vegetables than the mean of their respective age group populations (Su & Arab, 2006).

Nutrition interventions have been shown to be a cost-effective way to improve the health markers of seniors (Wunderlich, Bai, & Piemonte, 2010). By analyzing the demographic groups at the highest risk, such nutrition interventions can be better designed to suit the needs of these different demographic groups. The purpose of this study is to assess the relationship of food-related behaviors and demographic characteristics of nutritionally high-risk seniors. By exploring these relationships, nutrition interventions for the elderly can continue to improve as providers can tailor them towards the needs of different demographic groups.

## 2. Methods

### 2.1 The Study Instrument

The Nutrition Screening Initiative (NSI) was developed by the American Academy of Family Physicians, the American Dietetic Association, and the National Council on the Aging, Inc. as a quick and easy method to determine if an individual may be at nutritional risk (Bagley, 1998; Nutrition Screening Initiative, 1991). The content of the questions assesses seniors’ risk based on social and health based risk factors (Posner, 1993). The social and health risk factors were included in the NSI because they were determined to be the most prominent risk factors that are associated with poor nutrition. These seven risk factors were identified as: Inappropriate food intake, poverty, social isolation, dependency/disability, acute/chronic conditions, chronic medication use, and advanced age. Examining these risk factors, and scoring the health status, can help health providers to determine whether further nutrition intervention is appropriate without having to put seniors through costly and time-consuming tests (Sayhoun, 1999).

A supplemental questionnaire (SQ), based on the questions in the NSI checklist, was developed and administered to those who were identified as high-risk participants (having score of 6 or higher on the NSI checklist) as determined by the NSI. The purpose of the SQ was to obtain more specific information regarding the questions on the NSI that place them in the high-risk category. On the SQ the questions elaborated further regarding the number of meals they consume per day, servings of fruits and vegetables, servings of milk and milk products and if they could provide them, the reasons for their answers. The responses were analyzed in order to discover the common eating behaviors among different demographic groups of the older adults.

### 2.2 Sample Population and Survey Questions

The sample population for this study was 69 seniors with ages ranging from 56-91 years, who participated in government-sponsored congregate meal and nutrition programs in a single county in New Jersey. The sample size is small, as the nature of this study is preliminary. The congregate meal program in the county that the research was conducted, serves approximately 600 seniors per year; the above number represents those who were identified at high risk and also volunteered to complete the SQ. The survey was conducted in two quarters (half a year) of 2010. The number of participants (69) in this study represents about 24% of those who were identified as nutritionally high risk according to the NSI checklist during this time period. These individuals then completed the supplemental questionnaire (SQ), which provided an opportunity to explore in greater detail why these seniors were at nutritional risk. As a result of the small sample size, the sample population may not represent the older adult population in general. The purpose of this study was to improve the survey and to determine the factors that place this group in the nutritionally high-risk category. Participants were also asked to provide the following demographic information: gender, age, racial background, education, income range, marital status, number of people living in household, as well as their height and weight used for calculating their BMI (body mass index).

The questions on the SQ were coupled with multiple-choice answers, which attempted to hone in on why seniors were not practicing healthy nutritional behaviors such as not eating more than 2 meals per day or consuming more than 5 servings of fruits and vegetables a day (see Table 2). These responses were then analyzed with the demographic information in an effort to uncover significant relationships and trends between the responses and demographic characteristics. Montclair State University Institutional Review Board (IRB) approved the study protocol.

**Table 1:** Demographic characteristics of participants, N = 69

		N	%
**Age (year)**	55-70	21	30.4
	71-80	29	42
	80-90	16	23.2
	90+	3	4.3
**Age** Mean (SD)	74.29 (8.32)		
**BMI^a^**	20-25	18.0	27.3
	25.1-30	25.0	37.9
	31.1-35	15.0	22.7
	35.1-40	8.0	12.1
**BMI** Mean (SD)	28.35 (4.88)		
**Gender**	Male	17	24.6
	Female	52	75.4
**Race**	Caucasian	31	44.9
	Hispanic	9	13
	Asian	6	8.7
	African American	19	27.5
	Other	4	5.8
**Education**	Middle school	6	8.7
	High school	42	60.9
	College	20	29
	No formal education	1	1.4
**Marital Status**	Married	22	31.9
	Single	8	11.6
	Divorced	14	20.3
	Widowed	25	36.2
**Household**	Alone	38	55.1
	One other person	17	24.6
	Two other persons	9	13
	Three or more	2	2.9
	Four or more people	3	4.3
**Income Range ^b^**	$10,829 and less	3	16.7
	$10,830 - $14,569	4	22.2
	$14,570-$18,309	9	40
	$18,310-$22,049	1	5.6
	$22,050 and more	1	5.6

a : BMI (N= 66) was calculated by weight and height and not all participants responded to the question.

b : (N= 18), not all participants responded to this question.

**Table 2:** Breakdown of statistically significant responses

	Gender, N = 69											
**Eat alone, P= 0.02**	Male (N=17)					Female (N=52)						
I eat alone by choice	17.6					23.1						
No companions nearby	11.8					30.8						
I do not eat alone	52.9					44.2						
**Need Assistance, P = 0.011**	Male (N=17)					Female (N=52)						
Food Shopping	0					17.3						
Other	11.8					0						
Do not need assistance	88.2					82.7						

	**People living in household, N = 69**											
**Eat alone, P < 0.001**	Live alone (N=38)		1 other person (N=17)			2 other people (N=9)			3 other people (N=2)			4+ (N=3)
I do not eat alone	18.4		70.6			100			50			100
**Gained 10 Lbs, P=0.021**	Live alone (N=38)		1 other person (N=17)			2 other people (N=9)			3 other people (N=2)			4+ (N=3)
I am sedentary	0		5.9			0			0			33.3
I did not gain weight	92.1		94.1			100			50			66.7
Other	5.3		0			0			50			0

		**Marital Status, N = 69**												
**< 2 meals/day, P = 0.001**		Married (N = 22)			Single (N = 8)			Divorced (N = 14)			Widowd (N=25)	
I eat more than two meals per day		90.9			25			64.3			56	
Other		9.1			50			21.4			40	
**Money for food, P=0.034**		Married (N = 22)			Single (N = 8)			Divorced (N = 14)			Widowd (N=25)	
Cannot afford food		4.5			0			0			24	
Have enough money		90.9			87.5			100			76	

			**Education, N = 69**									
**5 Fruits/Veg per day, P < 0.001**				Middle School (N = 6)			High School (N=42)			College (N =20)		
I do not like fruits				0			2.4			0		
I do not like vegetables				16.7			0			0		
Not available for purchase				0			2.4			0		
Cannot afford				0			0			5		
I eat 5 servings				16.7			11.9			55		
Consumes, but less than 5 servings				50			66.7			40		
Occasionally				0			7.1			0		
**2 Milk/Milk Products per day, P < 0.001**				Middle School (N=6)			High School (N=42)			College (N =20)		
I do not like milk/milk products				16.7			7.1			5		
I do consume 2+ servings				33.3			28.6			35		
Consumes only 2 servings or less				16.7			23.8			15		
Cereal or Coffee only				16.7			26.2			15		
Other				16.7			4.8			10		
**Dental Problems, P = 0.007**				Middle School (N = 6)			High School (N=42)			College (N =20)		
I have no problems				100			66.7			100		
**Need Assistance, P = 0.046**				Middle School (N= 6)			High School (N=42)			College (N =20)		
Do not need assistance				66.7			83.3			95		
**I eat a nutritious diet, P = 0.019**				Middle School (N = 6)			High School (N = 42)			College (N =20)		
Strongly Agree				0			19			35		
Agree				66.7			42.9			55		

“No Education” not included in figure due to low participant number (N = 1)

### 2.3 Statistical Analysis

Descriptive statistics and Chi-Square tests were used for analysis using Predictive Analytics Software (PASW) (*www.ibm.com/PASW_Statistics*). This analysis was used to reveal the relationships between these variables and to identify the factors that can be effective in improving the interventions and to determine which risk factors were prominent among these groups. Chi-Square was performed to test for significance and the alpha level was set to < 0.05. For categories that have less than 5 expected values, Yates’ correction was applied.

## 3. Results

### 3.1 Demographic Characteristics of Participants

Table 1 presents the demographic characteristics of those high-risk seniors who participated in the study. The majority of participants were women (75.4%) and the mean age of all participants was 74.29 ± 8.32 years. The racial population in this study and the county where it took place were mostly Caucasians (44.9%) followed by African Americans (27.5%). Over half of the participants lived alone (55.1%) and 68.1% were not married at the time of the study. The majority of participants had received a high school education (60.9%). Some participants chose not to provide certain demographic information such as income level (N=18) and height and weight and therefore the number of participants is lower than 69 in these categories (N = 66). The BMI was calculated for 66 participants for whom the height and weight were recorded. Those participants who chose to provide their income fell within or under the poverty line. Most participants were overweight, with calculated BMI of 28.4 ± 4.9.

### 3.2 Overall Responses

**Figure 1:**
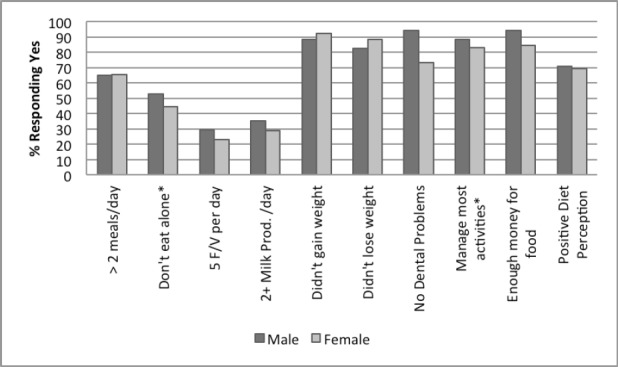
Participant responses to NSI checklist by gender, N = 69 a: NSI: Nutrition Screening Initiative *P < 0.05 **P<0.01

**Figure 2:**
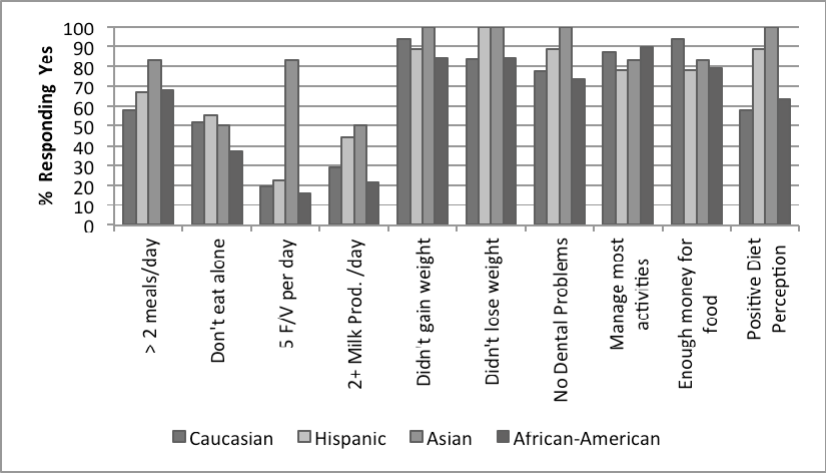
Participant responses to NSI checklist by race, N = 69 “Other” not included in Figure due to low participant number (N = 4)

**Figure 3:**
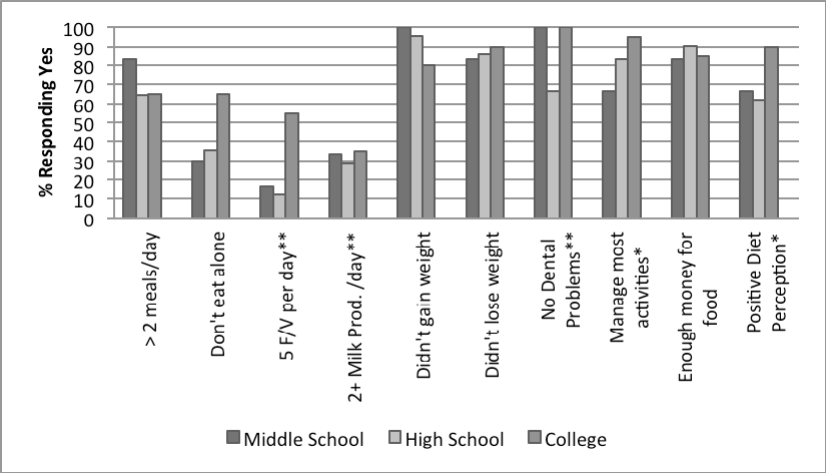
Participant responses to NSI checklist by education, N = 69 “No Education” not included in figure due to low participant number, (N = 1) *P < 0.05 **P<0.01

**Figure 4:**
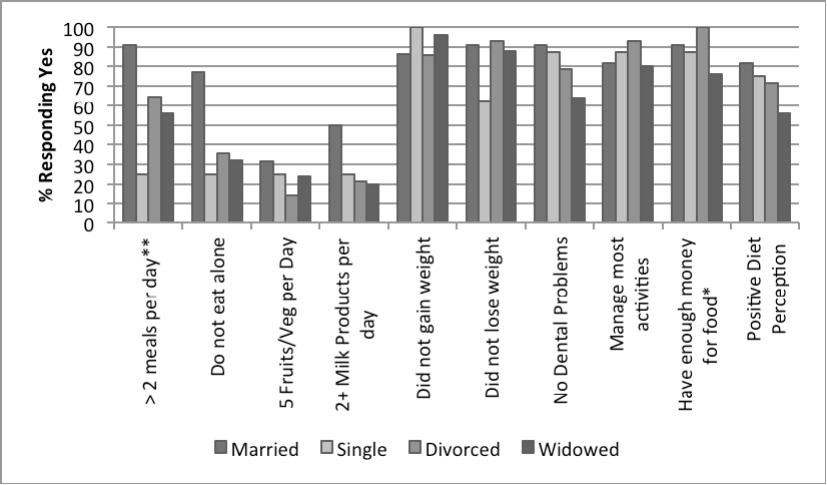
Participant responses to NSI checklist by marital status, N = 69 *P < 0.05 **P<0.01

**Figure 5:**
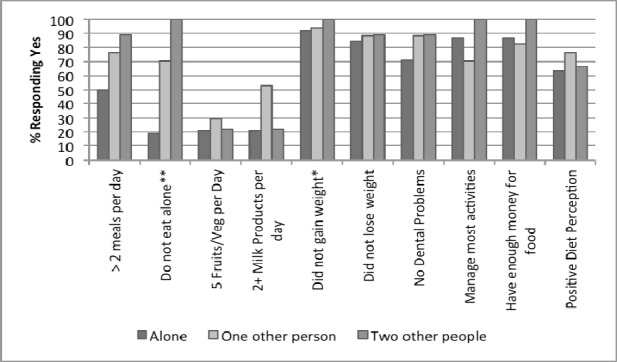
Participant responses to NSI checklist by number of people in household, N = 69 *P < 0.05 **P<0.01

Figures 1-5 illustrate the basic responses to the NSI checklist questions. These figures break down the responses by gender, racial background, education, marital status, and the number of people in the household. Both genders answered most of the questions in a similar fashion, as shown in Figure 1, except for the questions if they are eating alone and if they can manage most activities (see Table 2). More women were likely to report eating alone (p≤0.05) and men believed they could manage most activities (p≤0.05).

Figure 2 includes the responses by racial groups. Asians practiced healthy food behaviors and seemed to be the closest to optimal nutritional status. They ate more than 2 meals per day (83.3 %) and reported daily consumption of 5 or more servings of fruits and vegetables (83.3%). On the other hand, other racial groups reported consuming less fruits and vegetables and milk and milk products on a daily basis. For example, (19.4%) of Caucasians reported daily consumption of 5 or more servings of fruits and vegetables and (29.0%) reported consuming milk and milk products, whereas (15.8%) and (21.1%) respectively of African Americans and Hispanics did so.

Figure 3 indicates that with higher education, food behaviors and diet perception improve significantly. College educated individuals practiced healthier eating with significantly higher numbers of them eating 5 servings or more of fruits and vegetables (55%) and 2 or more servings of milk and milk products (35%) (p≤0.01).

The responses by marital status indicate that married people ate 2 or more meals/day (90.9%) and had a more positive diet perception (81.1%) as shown in Figure 4 (p≤0.01). Interestingly, the divorced individuals reported having enough money for food (100%) when compared to those who are widowed (76%) (p≤0.05). Figure 5 reveals that an additional one or two persons in the household improved overall nutrition behavior and health of the participants.

### 3.3 Reponses to the Supplemental Questionnaire

A supplemental questionnaire was administered to identify the explanation for eating behaviors among different demographic groups that contributed to the high-risk status.

Table 2 displays the participants’ responses to the questions on the supplemental questionnaire that the Yate’s corrected Chi-Square tests revealed were statistically significant. Some possible responses were not included for readability if there was a low response rate. The purpose was to highlight those responses that seemed the most salient. Participants’ responses were compared between demographic characteristics.

Women were more likely to eat alone (55.8%) when compared to men (47.1%). More women (30.8%) reported that they had no eating companions nearby compared to (11.8%) of men. Women were also more likely to eat alone by choice (23.1%) compared to men (17.6%), (p = 0.02). Men were also less likely to need assistance with their daily food related activities (88.2%). The remainder did not specify what they needed assistance with (11.8%). Participants were asked to provide reasons why they are eating alone. Women were more likely (30.8%) to report “no companions nearby” than men (11.8%). Also more women (21.1%) said they eat alone “by choice” vs. (17.6%) of the men.

Education level was a major contributor to change in the eating behaviors among older adults. The participant’s level of education produced the greatest amount of statistically significant responses among all of the demographic categories. Fifty five percent of those participants who graduated college consumed 5 servings of fruits and vegetables per day compared to 11.9% of high school graduates and 16.7% of middle school graduates, (p < 0.001). There was little variance between the amounts of milk or milk products consumed. Consumption, however, was significantly different among the responses regarding the reasons why (p < 0.001). College and middle school graduates reported the least amount of dental problems (100%). This may have been due to a greater income and better dental coverage over the participant’s lifetime in regard to college graduates, however, this could not be confirmed. College graduates were the least likely to need assistance with their everyday food related activities (95%), while 16.7% of middle school and 14.3% of high school graduates needed assistance in food shopping, (P = 0.046). Finally, college graduates had the most positive diet perceptions with 90% of them agreeing or strongly agreeing that they ate a nutritious diet compared to 66.7% of middle school graduates and 61.9% of high school graduates

Those participants who were married were the most likely to consume greater than 2 meals per day (90.9%) compared to 25% of those who were single, 64.3% of those who were divorced, and 56% of those who were widowed (P < 0.001). This was consistent with the observed trend in Figure 3 that shows that participants who were married were less likely to eat alone. Interestingly, those who were divorced reported being the most likely to have enough money for food (100%) compared to 76% of those who were widowed (P = 0.034). Although there is no clear-cut explanation for this, the researchers speculate that this trend may be a result of alimony payments for the divorced and no substitution for lost income for those that were widowed.

The number of people in the household made a difference in the responses regarding the sedentary life style and weight gain. The majority of the participants reported they are not following a sedentary life style and reported not to have gained 10 unwanted pounds over the previous 6 months (P= 0.021).

As one might imagine, those seniors living alone were more likely to eat in isolation. Only 18.4% of those seniors living alone reported that they did not eat in solitude. A majority of these participants (44.7%) reported that they ate alone because they had no companions nearby. The only other participant group to report that they had no companions nearby was those that lived with one other person (5.9%). Those living with 2 other people were the least likely to eat alone with 100% of participants reporting that that they ate with companions, (P < 0.001).

Although there were no significant P-values in the race/ethnicity category, there were some trends that were observed. As previously stated, the Asian population had very positive responses in comparison to the other ethnicities. They were the most likely to consume 5 servings of fruits and vegetables (83.3%) as well as milk or milk products (50%; See Figure 2).

## 4. Discussion

Many of the preliminary results obtained in this study are consistent with the scientific literature while some warrant further exploration:

### 4.1 Gender

This study found that males were less likely to eat alone and also to need assistance with their daily activities (Figure 1 & Table 2). One of the limitations of this study was that the female population greatly outnumbered the male population, thereby limiting the degree of significant information gleaned from this group (Table 1). Therefore, the results of this preliminary study may not be reflective of gender differences across a larger population. Some previously published studies have found a gap between males and females in regard to their nutritional risk (Quandt & Chao, 2000), particularly found that females possess a greater risk of malnutrition than males. Such results are consistent with this study. On the other hand, some studies have found no significant differences among gender and nutritional status (Sahyoun & Basiotis, 2001). Other studies have found gender to be a determining factor in healthy dietary behaviors such as fruit and vegetable consumption (Baker & Wardle, 2003; Lallukka et al, 2010).

Because literature on gender is inconsistent, the authors hypothesize that gender in the elderly may have a significant relationship to other risk factors such as marriage. Since females have longer life spans (US Census Bureau, 2012), they are more likely to be widowed and thereby be single. This in turn puts them at greater nutritional risk as discussed on the previous section because they are more likely to be living and eating alone.

### 4.2 Marital Status

The findings of this study indicate that those seniors who lived alone were significantly less likely to eat with other companions (Figure 5 & Table 2). Other studies have shown that eating together with companions increases food consumption in older adults (Wright, Hickson, & Frost, 2006). Links have also been shown between marital status and poor food intake with unmarried participants having the greatest risk of being insufficiently nourished (Sahyoun & Basiotis, 2001). Marital status (Figure 4 & Table 2) was also an indicative factor of food consumption in that those participants who were not married were significantly less likely to eat more than two meals per day. Since the elderly population is at a high risk of malnutrition (Dunne & Dahl, 2007), it can be implied from the results of this study that marital status and household status are indicative factors that may influence food consumption and eating behaviors among older adults. Furthermore, those participants who are not married and/or living alone may be at the greater nutritional risk.

### 4.3 Education

In this particular group, education was an indicator of several food behavior categories which may have contributed to placing the older adults at nutritional risk (Figure 3 & Table 2). The findings of this study are consistent with recent and past studies that have shown links between lower levels of education and inadequate food consumption and less optimal intake of nutrients (Sahyoun & Basiotis, 2001; Bianchetti, Rozzini, Carabellese, Zanetti, & Trabucchi, 1990). Higher levels of education in the elderly have also been associated with better nutritional knowledge and a more positive perception of health and nutrition (Lin & Lee, 2005). These results are also consistent with the findings of this study. Slightly outside the realm of this study, education levels of seniors have even been shown to have impacts on senior’s food preferences, a finding worth noting for those designing nutrition intervention programs for the elderly (Spangler & Pettit, 2003).

### 4.4 Race

There was no statistically significant relationship between race and any of the nutritional risk categories in this study. However, this may be due to the small sample size (N = 69) and the disproportionate distribution of different races. Some trends, however, were noted, as the Asian population seemed to perform better than the other races in several important categories such as meal frequency, fruit and vegetable consumption, and diet perception (Figure 2). The academic literature shows that race can play a significant role in overall nutrition status of the elderly (Weatherspoon, Worthen, & Handu, 2004; Marshall et al., 1999). Studies have even shown that race can even be related to adequate levels of micronutrients in the elderly such as B vitamins folate and B12 (Kim, Tse, & Boudreu, 2002). Recent research has shown relationships between vegetable consumption in relationship to race/ethnicity and distress levels (Kiviniemi et al., 2011). The authors believe that although race was not found to be statistically significant in this study, it is a demographic characteristic that should be considered for future studies and nutrition interventions for the elderly.

## 5. Limitations

A main constraint of the study was that a limited number of participants completed the survey at the time period the study was conducted. This was the first field test of the SQ partially designed to determine which areas need improvement. However, these preliminary results indicate that there were strong trends among demographic information such as gender, marital status, education, and household status.

## 6. Conclusion

As more and more Americans enter their senior years they will be confronted with various obstacles that pose a threat to their health. Understanding the different risk factors and causes of poor nutrition in seniors with different backgrounds is essential to their longevity and wellbeing. Considering the findings of this study, as well as previous studies in the scientific literature, it is important to continue exploring the relationships between demographic characteristics of the elderly and their nutritional risk. By fully understanding these relationships, health professionals can continue to improve care and design more effective and cost-efficient nutrition intervention programs.
